# The Sino-Himalayan flora evolved from lowland biomes dominated by tropical floristic elements

**DOI:** 10.1186/s12915-023-01746-4

**Published:** 2023-10-31

**Authors:** Yun Liu, Yang-Jun Lai, Jian-Fei Ye, Hai-Hua Hu, Dan-Xiao Peng, Li-Min Lu, Hang Sun, Zhi-Duan Chen

**Affiliations:** 1 State Key Laboratory of Plant Diversity and Specialty Crops, Institute of Botany, Chinese Academy of Sciences, 20 Nanxincun, Xiangshan, Beijing, 100093 China; 2 China National Botanical Garden, Beijing, 100093 China; 3 University of Chinese Academy of Sciences, Beijing, 100049 China; 4 State Key Laboratory of Biocontrol, School of Ecology, Shenzhen Campus, Sun Yat-Sen University, Shenzhen, 518107 China; 5 Key Laboratory of Biodiversity and Biogeography, Kunming Institute of Botany, Chinese Academy of Sciences, Kunming, 650204 China; 6 Sino-Africa Joint Research Centre, Chinese Academy of Sciences, Wuhan, 430074 China

**Keywords:** Asian monsoon, Endemism, Evolutionary history, Floristic elements, Assembly process, Uplift of Himalaya, Mountain biodiversity, Sino-Himalayan flora

## Abstract

**Background:**

The Sino-Himalayan flora harbors highly diverse high-elevation biotas, but our understanding of its evolutionary history in temporal and spatial dimensions is limited. In this study, we integrated a dated phylogenetic tree with comprehensive species distribution data to investigate changes over time and space in floristic elements, including the tropical, Tethys, northern temperate, and East Asian floristic elements, across the entire Sino-Himalaya and its three floristic regions: the Yunnan Plateau, Hengduan Mountains, and East Himalaya regions.

**Results:**

Our results revealed that the Sino-Himalayan flora developed from lowland biomes and was predominantly characterized by tropical floristic elements before the collision between the Indian subcontinent and Eurasia during the Early Cenozoic. Subsequently, from the Late Eocene onwards, the uplifts of the Himalaya and Hengduan Mountains transformed the Sino-Himalayan region into a wet and cold plateau, on which harsh and diverse ecological conditions forced the rapid evolution of local angiosperms, giving birth to characteristic taxa adapted to the high altitudes and cold habitat. The percentage of temperate floristic elements increased and exceeded that of tropical floristic elements by the Late Miocene.

**Conclusions:**

The Sino-Himalayan flora underwent four significant formation periods and experienced a considerable increase in endemic genera and species in the Miocene, which remain crucial to the present-day patterns of plant diversity. Our findings support the view that the Sino-Himalayan flora is relatively young but has ancient origins. The three major shifts in the divergence of genera and species during the four formation periods were primarily influenced by the uplifts of the Himalaya and Hengduan Mountains and the onset and intensification of the Asian monsoon system. Additionally, the temporal patterns of floristic elements differed among the three floristic regions of the Sino-Himalaya, indicating that the uplift of the Himalaya and surrounding areas was asynchronous. Compared to the Yunnan Plateau region, the East Himalaya and Hengduan Mountains experienced more recent and drastic uplifts, resulting in highly intricate topography with diverse habitats that promoted the rapid radiation of endemic genera and species in these regions.

**Supplementary Information:**

The online version contains supplementary material available at 10.1186/s12915-023-01746-4.

## Background

**Fig. 1:**
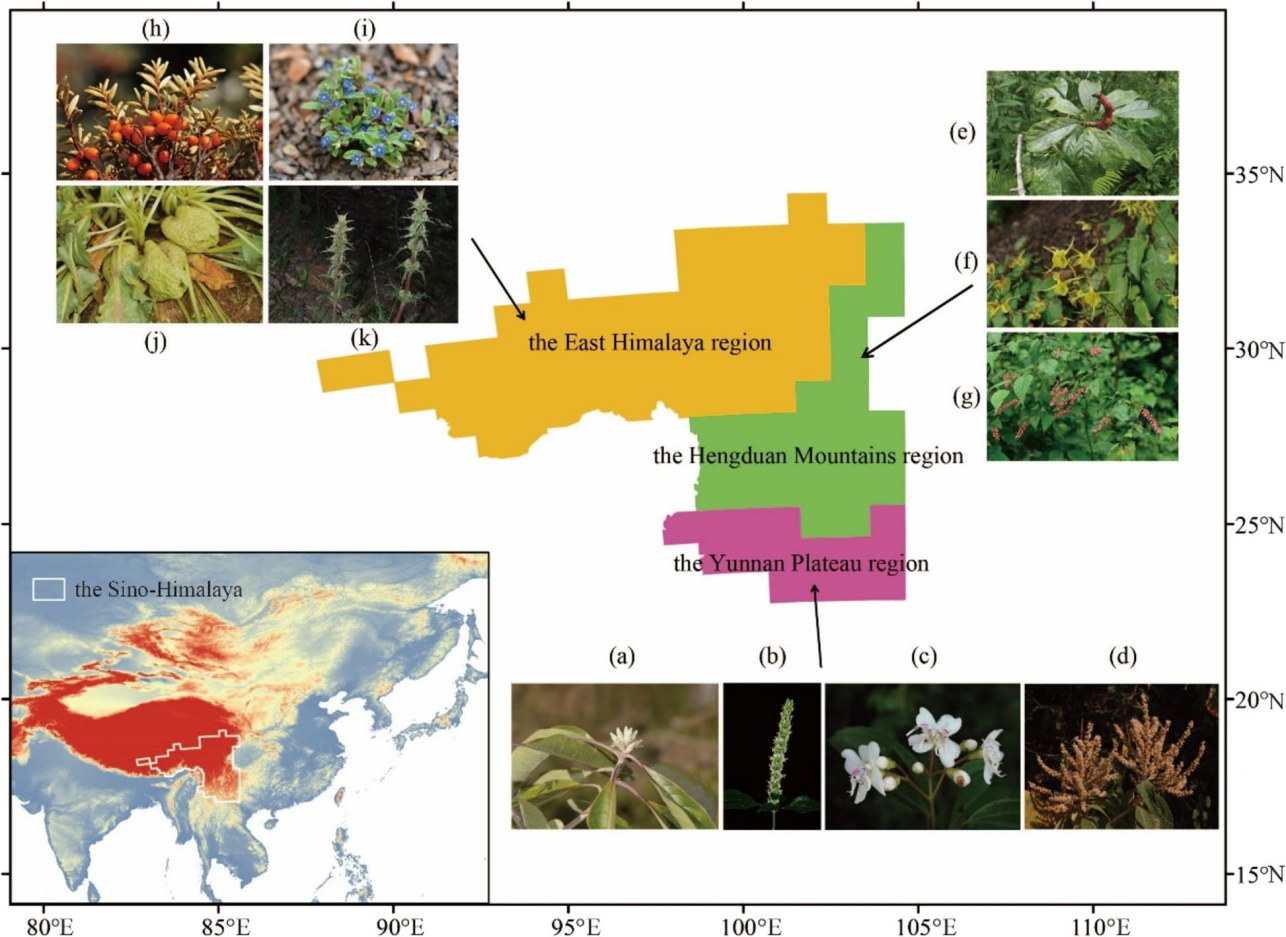
Range of the Sino-Himalaya and its three floristic regions based on Liu et al. [4]. The three floristic regions, the Yunnan Plateau region, Hengduan Mountains region, and East Himalaya region are indicated in pink, green, and yellow, respectively. Photos show representative extant species for each floristic region: **a** *Leucomeris decora*, **b** *Eurysolen gracilis*, **c** *Cyphotheca montana*, **d** *Colebrookea oppositifolia*, **e** *Oyama wilsonii*, **f** *Epimedium davidii*, **g** *Neillia thibetica*, **h** *Hippophae tibetana*, **i** *Microula pustulosa*, **j** *Przewalskia tangutica*, **k** *Morina kokonorica*. Photo credits: **a**, **h** Qinwen Lin; **b**, **g**, **i**, **j** Xinxin Zhu; **c**, **d**, **e**, **k** Bing Liu; **f** Yechun Xu

Mountains contribute significantly to the biodiversity of the Earth [1, 2], yet the causes behind their remarkable species richness, known as “Humboldt’s enigma,” have puzzled scientists for two centuries [1–3]. The Sino-Himalaya region, one of the world’s most biodiverse mountainous areas, is renowned for its rich temperate flora, which has been categorized into three floristic regions: the Yunnan Plateau, Hengduan Mountains, and East Himalaya regions (Fig. 1) [4]. With 17,611 species, 2,163 genera, and 222 families of angiosperms, it is one of the world’s most species-rich plant biodiversity conservation hotspots [4–7]. However, studies on the formation of this exceptionally species-rich flora are limited [7]. Investigating the evolutionary history of this flora is crucial for understanding mountain biodiversity, which is necessary for informed biodiversity conservation efforts in the face of accelerated global changes.

The Sino-Himalayan flora experienced significant changes due to the collision between the Indian subcontinent and Eurasia, which resulted in the retreat of the Tethys Sea during the Paleogene and the uplifts of the Himalaya and Hengduan Mountains [8, 9]. This geological transformation led to the development of diverse flora through colonization, *in-situ* diversification, and local extinction. The Sino-Himalayan flora consists of several floristic elements such as the laurophyll ‘paleotropical geoflora’ in the Paleogene, the Mediterranean xerophyte flora in the Neogene, the Arctic-Tertiary flora, and the modern northern temperate flora [8–10]. The migration of floristic elements from other floras served as a crucial source for the Sino-Himalayan flora. For example, genera of Lauraceae (e.g., *Cinnamomum*, *Litsea*, and *Neolitsea*) and Fagaceae (e.g., *Castanopsis*, *Cyclobalanopsis*, and *Quercus*) found in the Sino-Himalayan flora are descendants of the Tethyan flora that existed from the Late Cretaceous to the Early Paleogene [9]. Additionally, some taxa (e.g., *Astilbe*, *Rodgersia*, *Saxifraga*, and *Triosteum*) originated from the boreal or Arctic-Tertiary flora [9, 10]. *In-situ* diversification, triggered by the uplifts of the Himalaya and Hengduan Mountains and intensification of the Asian monsoon, was also a significant source of the recent evolution in the Sino-Himalayan flora [11, 12]. Studies have shown that the extensive uplifts of the Himalaya and Hengduan Mountains played an essential role in the diversification of *Cyananthus* and *Saussurea* [13, 14]. Reconstructing the spatiotemporal changes in various floristic elements can help us understand the evolution of the Sino-Himalayan flora.

The Sino-Himalaya harbors high endemic plant diversity, particularly in the Hengduan and East Himalaya Mountains [15]. The Tibetan Plateau alone hosts 3,673 endemic seed plant species, including *Aconitum bracteolatum*, *Allium nanodes*, *Cremanthodium medogense*, *Rheum nobile*, and *Terminalia franchetii* [16, 17]. The Himalaya and Hengduan Mountains harbor at least 90 endemic genera, such as *Acronema*, *Cyclorhiza*, and *Solms*-*laubachia* [18]. The complex topography caused by orogeny, such as the uplifts of the Himalaya and Hengduan Mountains, and climate changes, such as the onset of the Asian monsoon system, may promote high plant endemism via mechanisms like allopatric speciation through geographic isolation and climatic oscillations [17, 19–22]. The spatiotemporal patterns of endemic taxa are critical to biogeography because they capture unique facets of biodiversity, reflecting the floristic features and evolutionary dynamics of a specific flora in response to geological events and climate changes in different geological periods [21, 23].

Considerable progress has been made in researching the biodiversity and biogeographic patterns of the Himalayan and Hengduan Mountains regions since the concept of the Sino-Himalayan flora was introduced by Ward in 1927 [24]. Previous studies have mainly focused on taxonomic diversity, such as similarities in families, genera, and species among floras [8–10, 16, 25]. However, with the advancement of molecular systematics, dated phylogenetic trees that depict the phylogenetic relationships of lineages and provide information about speciation, extinction, and migration can offer new evidence for inferring the origin and diversification of taxa in the temporal dimension [26, 27]. In the past two decades, many studies have examined the origin and dispersal of specific taxa related to the Sino-Himalayan flora using molecular biogeographic methods [14, 19, 22, 28–33], while others have investigated the spatiotemporal pattern of flora using representative taxa through meta-analysis [11, 12, 34]. However, different taxa with specific biological and ecological characteristics may exhibit varying responses to geological events and climate changes, resulting in different evolutionary histories and incongruent biogeographic patterns among lineages [35, 36]. Therefore, knowledge of the timing and routes of origin and dispersal based on a single taxon or a few taxa may be insufficient and could limit our understanding of the spatiotemporal biogeographic patterns of an entire biota [37, 38].

The regional tree of life is a useful tool for investigating biogeographic regions and the processes that shape them [39]. It integrates individual taxon histories into shared biota histories and is typically reconstructed through intensive sampling of the regional species pool [40]. Using dated phylogeny and spatial data, we can estimate the divergence time and evolutionary rates of a flora and integrate paleogeological and paleoclimatic data to elucidate the underlying ecological and historical factors of floristic biodiversity patterns [39]. Recent studies have reconstructed the evolutionary history of numerous biotas using robust regional dated phylogenies and detailed species distributions. These investigations provide a useful approach for exploring the evolutionary history of the Sino-Himalayan flora [17, 40–47].

In this study, we aimed to investigate the assembly processes of the Sino-Himalayan angiosperm flora in different geological periods, using dated phylogeny and species distribution data. Specifically, we had the following objectives: (i) examine the spatiotemporal divergence patterns of the Sino-Himalayan flora at different taxonomic levels (families, genera, and species); (ii) assess the spatial and temporal changes in various floristic elements over time; (iii) compare the evolutionary history of floras across the three floristic regions of the Sino-Himalaya; and (iv) explore the factors that drive the distribution patterns of extant floras of the Sino-Himalaya as a whole and within its three floristic regions.

## Results

### Divergence patterns of families, genera, and species over geological time

**Fig. 2:**
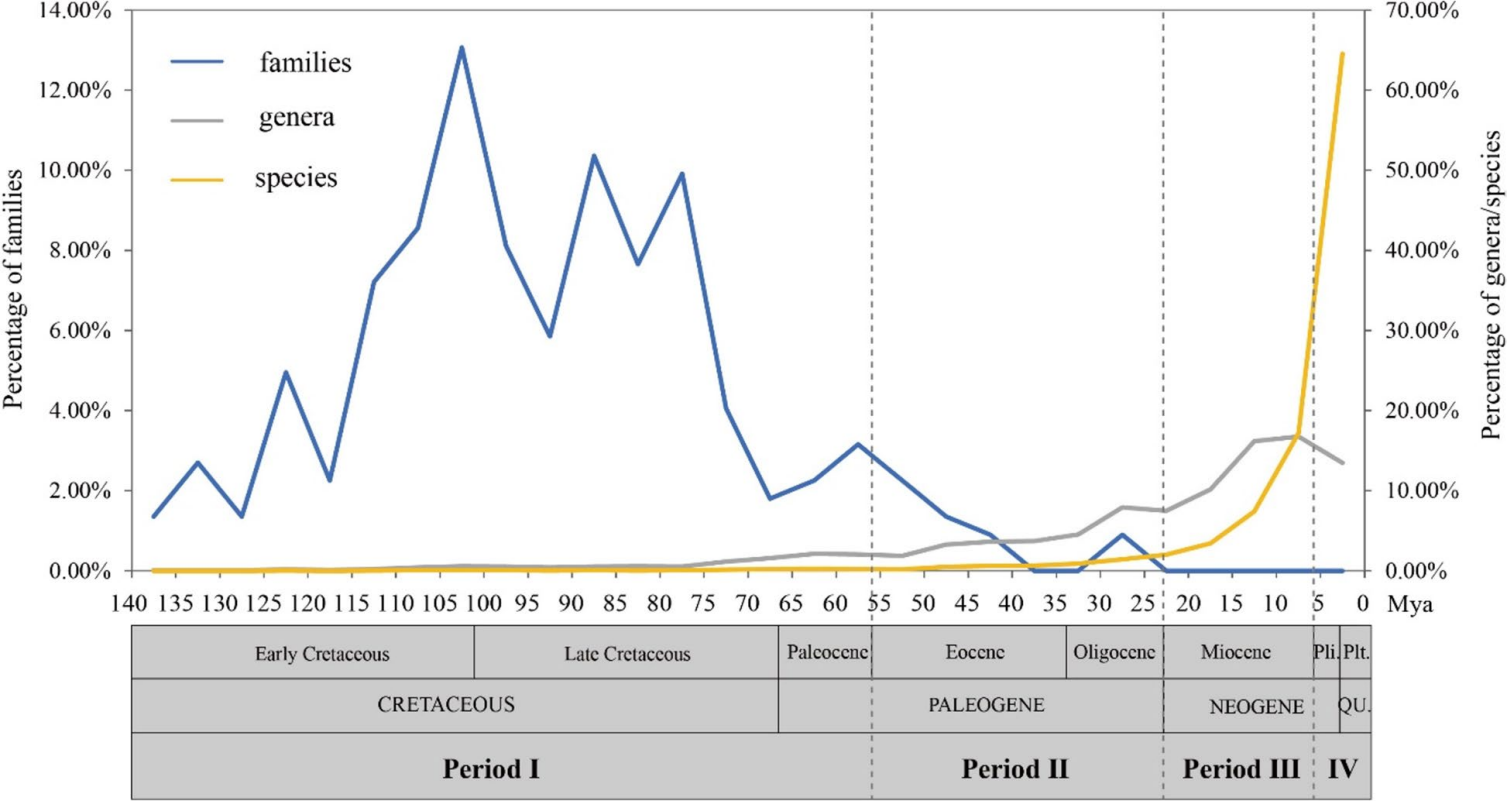
Divergence patterns of angiosperm families, genera, and species at different geological times, and four periods and three major shifts of the evolutionary history of the Sino-Himalayan angiosperm flora. Period I (Cretaceous–Paleocene) marks the onset of flora characterized by the emergence of angiosperm families. Period II (Eocene–Oligocene) marks the gradual development of flora characterized by the formation of angiosperm genera. Period III (Miocene) is characterized by the rapid divergence of angiosperm genera. Period IV (Pliocene–Quaternary) is characterized by the rapid divergence of angiosperm species. The three gray vertical dotted lines represent three major shifts in genera and species divergence. Blue, gray, and yellow lines represent the percentages of families, genera, and species, respectively, that diverged during each five-million-year period. Pli., Pliocene; Plt., Pleistocene; QU., Quaternary

Divergence time estimates indicate that Sino-Himalayan angiosperm families began to diverge in the Early Cretaceous (Fig. 2). Statistical analysis revealed that 88% of angiosperm families diverged during the Cretaceous, with the Late Cretaceous witnessing the highest number (107 families, 48%; Fig. 2; Additional file 1: Table S1). No family diverged after the Oligocene. Approximately 85% of Sino-Himalayan angiosperm genera had occurred by the end of the Miocene, with the highest percentage (46%) diverging during the Miocene (Fig. 2; Additional file 1: Table S2). At the species level, 93% of angiosperm species have diverged since the Miocene, with nearly half (47%) diverging during the Quaternary (Fig. 2; Additional file 1: Table S3).

The angiosperm divergence patterns over geological time in the three floristic regions of the Sino-Himalaya followed similar trends to those in the entire region (Additional file 1: Fig. S1; Tables S1–S3).

### Distribution types and floristic elements over geological time

Distribution types 2, 7, 8, and 14 were the main distribution types in the Sino-Himalayan flora (Additional file 1: Fig. S2a). The percentage of diverged genera with distribution types 1–3, 8, and 12 decreased, while that of genera with distribution types 7, 10, 11, and 13–15 increased over geological time (Additional file 1: Fig. S2b, c).

**Fig. 3:**
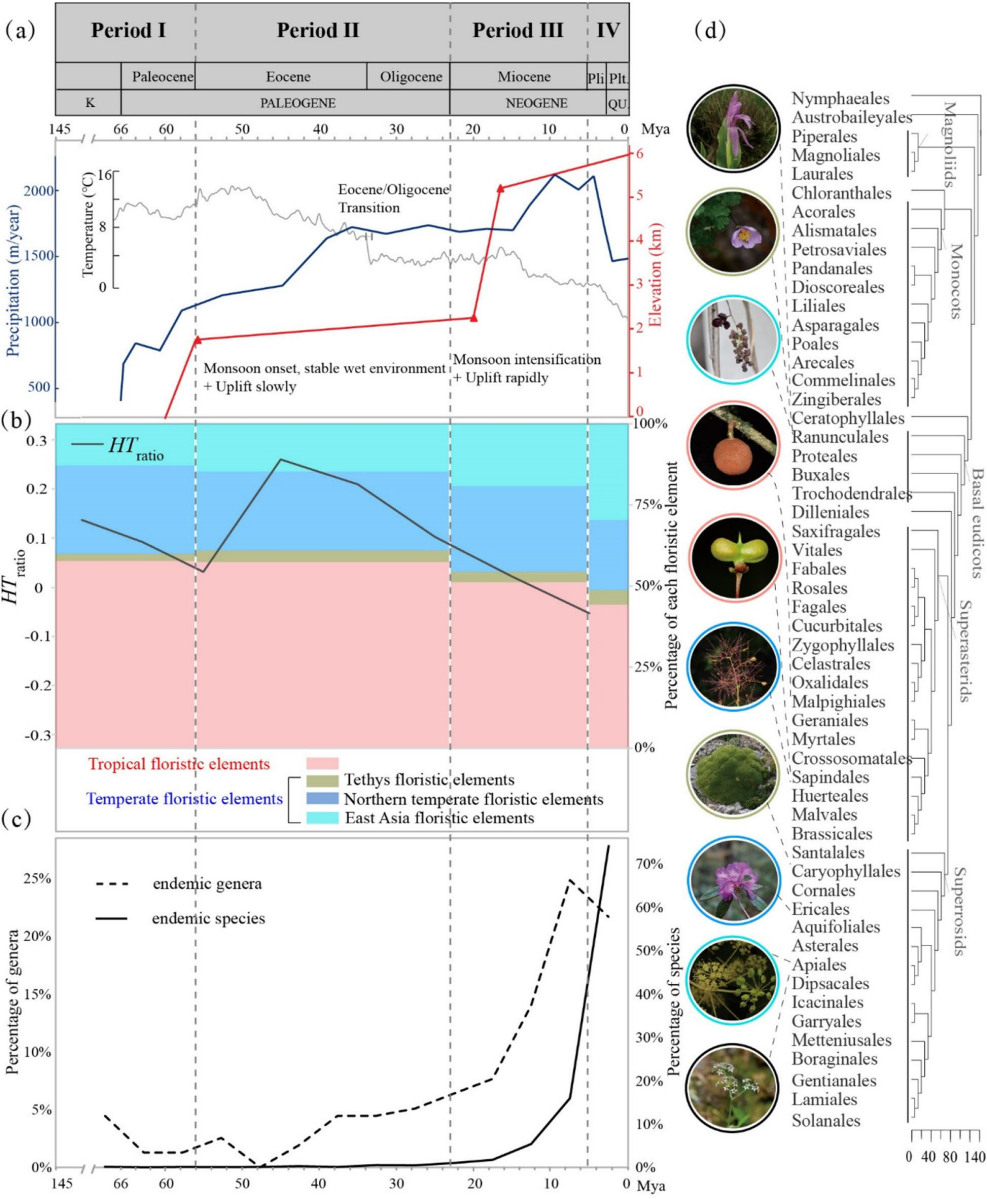
Temporal changes in floristic elements and endemic taxa of the Sino-Himalayan angiosperm flora in relation to geologic events and climate changes during four formation periods. **a** Major factors driving the establishment of the Sino-Himalayan angiosperm flora. The four formation periods and three major shifts are shown in Fig. 2. The red line refers to the Himalaya uplift, which began in the Early Paleocene, and accelerated in the Miocene (adapted from [48]). The blue line presents monsoon conditions indicated by the modeled mean annual precipitation for each geologic stage (modified from [49]). The gray curve represents the estimated deep ocean temperature as an indicator of global climate changes (according to [50]). K, Cretaceous; Pli., Pliocene; Plt., Pleistocene; QU., Quaternary. **b** The percentage of genera belonging to four floristic elements diverged during the four formation periods in the Sino-Himalayan flora is shown in histograms with different colors. The black line represents the ratio of tropical (*H*) to temperate (*T)* floristic elements (*HT*_ratio_). *HT*_ratio_ = (*H*-*T*)/(*H* + *T*). *HT*_ratio_ < 0, temperate floristic elements are dominant in the studied period; *HT*_ratio_ > 0, tropical floristic elements are dominant in the studied period. **c** Changes in divergence of endemic genera and species in the Sino-Himalayan angiosperm flora over time. The percentage of diverged endemic genera and species is shown by a dotted line and solid line, respectively. **d** Ordinal time-tree with major angiosperm clades indicated. The photos of extant plants show the representative taxa of each floristic element of the Sino-Himalayan flora. The five differently colored borders of the photos correspond to the four types of floristic elements in panel b; endemic taxa are indicated in black. From top to bottom, the genera in the photos are: *Roscoea*, *Paraquilegia*, *Akebia*, *Gynocardia*, *Delavaya*, *Cotinus*, *Thylacospermum*, *Rhododendron*, *Kalopanax*, and *Acronema*. Photo credits: *Paraquilegia* (Boka Li); *Akebia*, *Acronema* (Ze Wei); *Delavaya* (Xinxin Zhu), *Rhododendron* (Zi Wang)*.* Other photos are from Bing Liu

Until 10 million years ago (Mya), tropical floristic elements accounted for over half of the Sino-Himalayan angiosperm genera. However, temperate floristic elements eventually overtook after an increase from ~ 40 Mya onwards (Fig. 3b and Additional file 1: Fig. S3a). During the Paleogene, Pantropical (distribution type 2) and Tropical Asian (Indo-Malaysian) (distribution type 7) genera contributed the most to the tropical floristic elements (Additional file 1: Fig. S4a, b). The percentage of Pantropic (distribution type 2) genera that diverged fluctuated but showed an overall decreasing trend, while that of Tropical Asian (Indo-Malaysian) (distribution type 7) genera generally increased over geological time (Additional file 1: Figs. S2b, c, and S4b). The Tethys floristic elements showed the lowest percentage over geological time (Additional file 1: Fig. S4a). Their percentage increased significantly in the Eocene and peaked in the Pliocene (Additional file 1: Fig. S4a). The percentage of genera with Central Asian distribution (distribution type 13) increased, while that of genera with Mediterranean, West Asian to Central Asian distribution (distribution type 12) generally decreased over geological time (Additional file 1: Figs. S2b, c, and S4c). Since the Cenozoic, the northern temperate floristic elements had the highest percentage in the Miocene, and the genera with North Temperate distribution (distribution type 8) contributed the most to the temperate floristic elements (Additional file 1: Fig. S4a, d). The East Asia floristic elements had the highest percentage in the Pliocene, and the genera with East Asian distribution (distribution type 14) contributed the most to this element (Additional file 1: Fig. S4a, e).

**Fig. 4:**
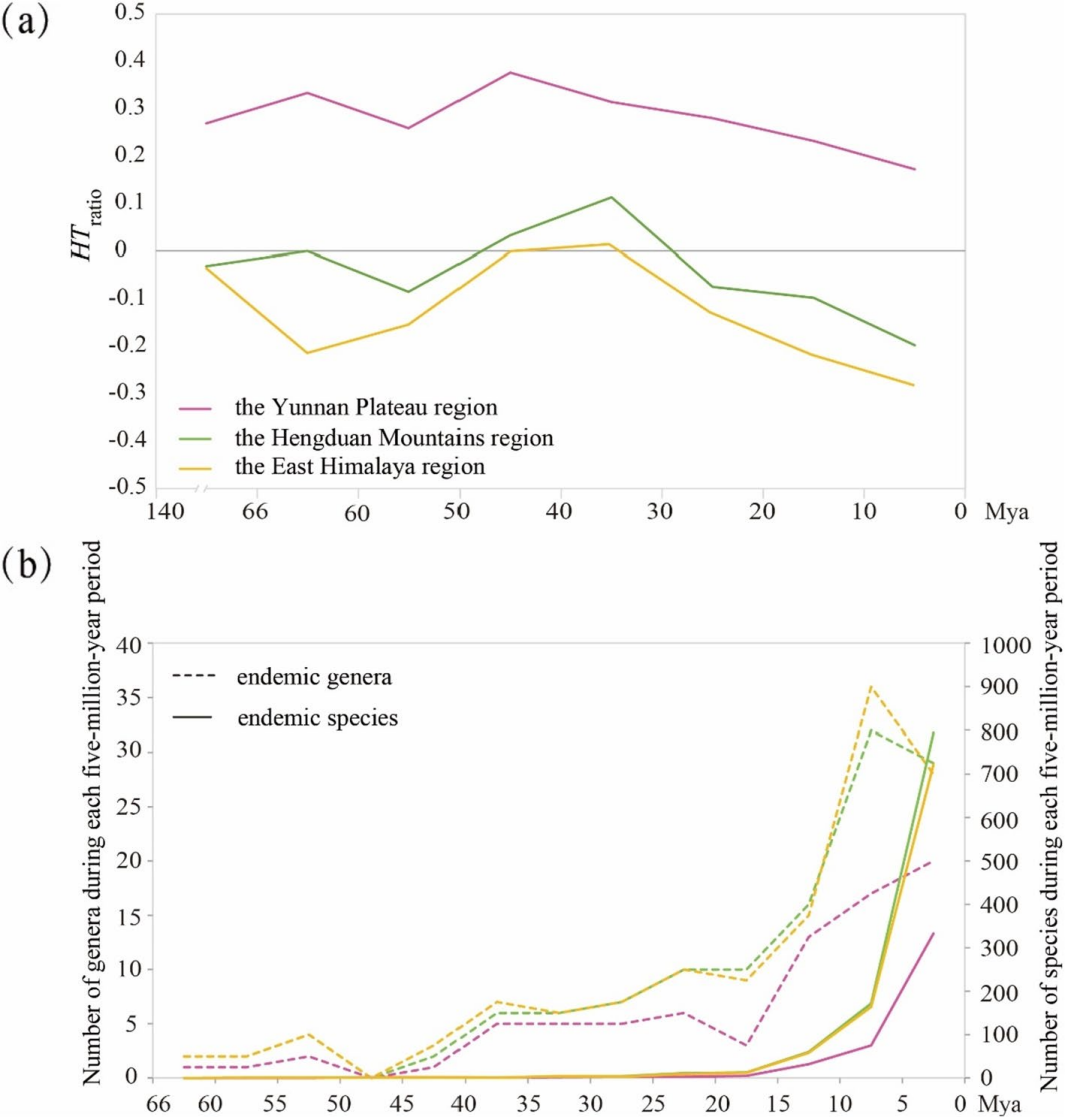
Temporal patterns of *HT*_ratio_ and endemic taxa of angiosperms in three floristic regions. **a** The ratio of tropical to temperate floristic elements is represented by the *HT*_ratio_. For *HT*_ratio_ definition, refer to Fig. 3. Lines with different colors represent three floristic regions (the Yunnan Plateau, Hengduan Mountains, and East Himalaya) within the Sino-Himalaya. **b** Changes in divergence of endemic genera and species in the three floristic regions over geological time. The number of diverged endemic genera and species are shown as dotted lines and solid lines, respectively

The temporal patterns of divergence for tropical and temperate floristic elements varied across the three floristic regions. The Yunnan Plateau region was dominated by tropical floristic elements throughout geological time, while the East Himalaya region was dominated by temperate floristic elements over the same period (Fig. 4a; Additional file 1: Fig. S3b, d). In the Hengduan Mountains region, tropical floristic elements were more abundant than temperate floristic elements between 50 and 30 Mya, while temperate floristic elements became dominant after 30 Mya (Fig. 4a; Additional file 1: Fig. S3c).

### Divergence patterns of endemic genera and endemic species over geological time

In the Sino-Himalayan angiosperm flora, there are 145 endemic genera and 1,213 endemic species (Additional file 1: Tables S2 and S3). The Yunnan Plateau region has 84 endemic genera and 458 endemic species, the Hengduan Mountains region has 132 endemic genera and 1,063 endemic species, and the East Himalaya region has 135 endemic genera and 979 endemic species (Additional file 1: Tables S2 and S3). Approximately 71% of the endemic genera in the Sino-Himalayan angiosperm flora have diverged since the Miocene, with the highest percentage of divergence occurring during the Miocene (~ 46%; Fig. 3c; Additional file 1: Tables S2 and S3). Approximately 98% of endemic species have diverged since the Miocene, and ~ 56% of endemic species diverged during the Pleistocene (Fig. 3c; Additional file 1: Tables S2 and S3).

The overall divergence of endemic genera has gradually increased since the Early Eocene (~ 50 Mya). The divergence rate of endemic genera increased significantly from the Miocene onwards, particularly after 20 Mya, and reached its peak between 10 and 5 Mya (Fig. 3c). The overall endemic species divergence tended to gradually increase from the Miocene onwards and peaked during the Pliocene–Quaternary period (Fig. 3c). The divergence rate of endemic species has significantly increased since the Miocene, especially after 10 Mya (late Miocene; Fig. 3c).

The divergence patterns over time of endemic genera and species in the three floristic regions were generally similar to those of the entire Sino-Himalaya region (Figs. 3c and 4b). However, there were some variations between the three regions during the same geological period. The divergence of endemic genera in the East Himalaya region decreased, while it remained constant in the Hengduan Mountains region between 40 and 30 Mya. In the Hengduan Mountains region, divergence was higher than in the other two regions between 25 and 20 Mya (Fig. 4b). The divergence rate of endemic genera in the East Himalaya and Hengduan Mountains regions increased considerably, and that in the former was much higher between 15 and 10 Mya, whereas it decreased in the Yunnan Plateau region (Fig. 4b). The number of diverging endemic genera in the Yunnan Plateau region slowly increased, while it declined in the other two regions from 5 Mya onwards. Moreover, the divergence of endemic genera and species in the Yunnan Plateau region was consistently the lowest in each geological period (Fig. 4b). The number of diverged endemic species in the Hengduan Mountains region exceeded that in the other two regions within the Sino-Himalaya over time (Fig. 4b).

## Discussion

### Four periods of the evolutionary history of the Sino-Himalayan flora

The Sino-Himalayan flora underwent four main formation periods, each characterized by distinct divergence patterns of taxa (families, genera, and species) and floristic elements, and coincided with fossil evidence (Figs. 2 and 3; Additional file 1: Fig. S4, Table S4).

Period I: Cretaceous to Paleocene (~ 145–56 Mya). The Sino-Himalayan flora emerged as the Tethys Sea receded south of Lhasa, leading to the development of the Sino-Himalayan angiosperm flora [8, 51]. The majority of angiosperm families (95%) and a few genera (10%) of the extant Sino-Himalayan flora occurred during this period. The flora was dominated by tropical floristic elements, with evergreen trees and shrubs such as Lauraceae, Fagaceae, and Arecaceae evolving in the region, along with some taxa in the subtropical and tropical mountains of East Asia such as *Aphananthe*, *Meliosma*, *Saurauia*, and *Symplocos* [8, 9]. The fossil records of *Laurophyllum* and *Symplocospollenites* presented in the Xigaze group (late Cretaceous) [52]. Genera with Pantropic and Tropical Asian (Indo-Malaysian) distributions were the most dominant in this period, and the percentage of genera with Tropical Asian (Indo-Malaysian) distribution showed a general upward trend in the subsequent periods (Additional file 1: Fig. S4b). This suggests an increase in floristic exchanges between the Sino-Himalayan flora and the Indo-Malaysian flora, possibly facilitated by island chains or peninsulas, drifting of India, clockwise rotation and displacement of the Simao-Lanping geoblock to the southeast, and the extrusion of the Indochina geological plate toward Southeast Asia [53–55]. The palynological assemblage of the Cuojiangding section in Zhongba [56] and the megafossils of northeastern India [57] indicated a tropical lowland environment. This suggests that the Sino-Himalaya region corresponding to the present-day Himalaya and Hengduan Mountains areas was low in altitude and had a tropical climate, which supported tropical lowland biomes. Moreover, the region was influenced by genera with tropical floristic elements that may have migrated from other floras, especially from the Pantropic and Tropical Asian (Indo-Malaysian) regions.

Period II: Eocene to Oligocene (~ 56–23 Mya). The collision of the Indian Plate and the Eurasian continent around 56 Mya led to the gradual uplift of the Tibetan Plateau [37, 58, 59]. During the Eocene to Oligocene period, there was a gradual divergence of all (28%) and endemic genera (23%) in the Sino-Himalayan flora.

During this period, the Sino-Himalayan flora was still mainly composed of tropical floristic elements, but the percentage of temperate floristic elements gradually increased, starting from ~ 40 Mya. The fossil floral composition from the Lawula Formation in Markam indicated climate and biotic changes from a subtropical/warm to a cool temperate climate in south-eastern Tibet during the latest Eocene [60]. Notably, the percentage of Tethys floristic elements, such as *Pistacia* (fossil record in Lunpola Basin) [61], *Plumbagella*, *Stephanachne*, *Thylacospermum*, *Trachydium*, and *Tricholepis*, increased from the Eocene onwards. The fossil communities were dominated by the xerophytic elements of *Ephedra-Nitraria* from the Yaxicuo Group in the Hoh Xil Basin [62] and the Gongjue Formation in the Nangqian Basin [63], indicating a dry climate during the Eocene. This may have been due to the aridification and cooling of the Sino-Himalayan climate caused by the retreat of the Tethys Sea and the uplifts of the Himalaya and Hengduan Mountains. Moreover, the contribution of genera with Central Asian distribution also increased, likely as a result of prolonged drought in Central Asia due to global cooling and uplift of the Tibetan Plateau. The aridification of the region may have created new habitats for speciation and facilitated vicariance events. Thus, the Sino-Himalayan flora was in a gradual development stage, characterized by dominant tropical floristic elements, but an increasing presence of temperate floristic elements, especially Tethys floristic elements.

Period III: Miocene (~ 23–5 Mya). Compared to other periods, the Miocene was a significant period in the divergence of genera and the development of the temperate floristic elements in the Sino-Himalayan angiosperm flora. Fossil records demonstrated that the Tibetan Region exhibited a pronounced temperate floristic composition, while the Yunnan Plateau and the eastern Himalayan Siwalik predominantly showcased subtropical and tropical elements [64–66]. During this period, there was a major burst of diversification, with almost 50% of the genera diverging, particularly the endemic genera (46%). Furthermore, there was a noticeable increase in the divergence rates of endemic genera and species. These supplied the majority of the Sino-Himalayan endemic elements, which subsequently diversified from the Miocene onwards, to create today's relatively high endemicity in the Sino-Himalaya [7]. The changes in climate and habitat in the Miocene are believed to have driven the formation of endemic genera, such as *Diplazoptilon*, *Dolomiaea*, and *Xanthopappus* [67].

During this period, the proportion of temperate floristic elements increased by 49%, with northern temperate floristic elements being the most abundant and East Asian floristic elements showing a significant increase. The uplifts of the Himalaya and Hengduan Mountains and climate cooling likely allowed the Tethyan flora to adapt to the alpine environment and gradually differentiate into northern temperate floristic elements [9]. The Arctic-Tertiary temperate flora also rapidly migrated southward due to a drop in temperature at middle to high latitudes in the northern hemisphere during the Miocene [10]. The mountainous region in the Sino-Himalaya provided wet and cool conditions, which facilitated the survival and differentiation of the Arctic-Tertiary temperate flora, leading to the development of northern temperate floristic elements and the formation of a modern center of distribution and diversification of genera, such as *Delphinium*, *Rhododendron*, and *Saxifraga*, in the Sino-Himalaya region [10, 32, 68–70]. Our results support the idea that the Miocene was a focal point for the development of the Sino-Himalayan angiosperm flora, similar to the herpetological fauna [71], characterized by a burst of genera, especially endemic genera, and a significant increase in temperate floristic elements.

Period IV: Pliocene to Quaternary (~ 5 Mya to present). From the Miocene, angiosperm species gradually flourished and reached their peak during the Pliocene to Quaternary period, with more than half of the endemic species originating during this period. The time delay between the explosion of genera and species could be attributed to the interval required for acquiring the necessary morphological, reproductive, and ecological innovations for species radiation.

During this period, the Sino-Himalayan angiosperm flora exhibited a higher proportion of temperate (56%) than tropical floristic elements. Among these, East Asian floristic elements such as *Clematoclethra*, *Euscaphis*, *Kalopanax*, *Liriope*, *Trochodendron*, and *Tubocapsicum* were the most abundant. This distribution pattern may have been influenced by changes in paleoclimate and/or orogeny, as suggested by studies on *Rodgersia* and *Stachyurus* [72, 73], possibly due to the uplift of the Himalaya and changes in the intensity of the monsoon system and Pleistocene climatic oscillations. Phylogeographic studies showed that several lineages (e.g., *Draba*, *Solms-laubachia*, *Rosa soulieana*) experienced a rapid and extensive range expansion in response to the Neogene plateau uplift and/or Quaternary climate fluctuations [17]. As a result, the Sino-Himalayan flora became characterized by highly diversified endemic species and a dominance of temperate elements during this period.

### Major factors driving the formation of the Sino-Himalayan flora

The proposal of Spicer [74] regarding the influence of the Himalayan rise and the Asian monsoon on species diversification in the region surrounding the Tibetan Plateau and Himalaya has been supported by recent studies on alpine flora diversification and herpetological phylogeographic analyses in the Himalaya [11, 71]. Our study revealed three significant shifts in biotic diversification during the evolutionary history of the Sino-Himalayan flora, which were strongly associated with the remarkable orogeny and intensity of the Asian monsoon system in the area during the Cenozoic (Figs. 2 and 3). Therefore, the evolution of the Sino-Himalayan flora was likely influenced jointly by the Himalayan uplift and the onset and intensity of the Asian monsoon (Fig. 3a), which not only facilitated uplift-driven *in-situ* diversification but also had an impact on biotic interchanges.

The first significant shift occurred at the transition from Period I to Period II (around 56 Mya), with the number of genera and the proportion of temperate floristic elements in the Sino-Himalayan flora increasing between 60 and 50 Mya, which coincided with the initial uplift of the Himalaya in the Paleocene (about 60 Ma) [48]. From the Eocene to the Oligocene, the gradual uplift of the Himalaya was accompanied by a gradual increase in the diversification of genera and the percentage of temperate floristic elements in the Sino-Himalayan flora. The onset and development of the Asian monsoon system, associated with the gradual uplift of mountains, created a stable and wet environment that stimulated plant diversification [75]. Furthermore, the number of temperate floristic elements increased significantly around 40–30 Mya, particularly in the Hengduan Mountains and East Himalaya regions, as a result of the uplift extension and cooler climate from the Eocene–Oligocene transition onwards (35–30 Mya) [14]. This stimulated the diversification of alpine plants and contributed to the expansion of alpine flora during this period [37, 76, 77].

The second major shift occurred during the transition from Period II to Period III (~ 23 Mya), marked by a significant increase in the diversification of genera and species, especially the endemic ones. The divergence rate of endemic genera and species rose sharply from the Miocene onwards, particularly during 20–15, 15–10, and 10–5 Mya. This remarkable diversification is believed to be driven by the uplift of the Himalaya, which began in the Miocene and caused a dramatic increase in elevation from ~ 2.3 km at the beginning of the Miocene to at least ~ 5.5 km by ~ 15 Mya, consequently impacting the intensity of the Asian monsoon system during this period [11, 12, 48]. This pattern agrees with the herpetological phylogeographic analyses by Xu et al. [71] and supports the stepwise model proposed by Ding et al. [48], in which the Himalaya initially grew slowly and gradually, but then rapidly during the Miocene (Fig. 3a).

The third major shift occurred at the transition from Period III to Period IV (~ 5 Mya), during which the number of genera decreased while species, especially endemic species, continued to diverge at high rates. This pattern may be attributed to the rapid uplift of the Himalaya and the intensification of the Asian monsoon system at this time. Many studies have shown that the Asian monsoon system intensified progressively due to Neogene mountain uplift, especially at 10–5 Mya, despite ongoing debate regarding its onset and intensity [37, 49, 78, 79]. The enhanced monsoon system provided greater moisture availability for sustaining vegetation and associated biota [35], as well as erosion through river incision, resulting in increased topographic relief [80]. These changes likely promoted the differentiation of microhabitats associated with elevational gradients and slope aspect, thereby increasing the potential for local adaptation, ecological divergence, and lineage diversification [11, 81].

The floristic development of the three floristic regions within the Sino-Himalaya did not synchronize completely in response to geological and climate changes, which is of particular interest. The endemic genera in the Hengduan Mountains region began to diverge more significantly than those in the other two regions from the Eocene–Oligocene Transition (35–30 Mya), due to the gradual narrowing of the Tethys caused by the continuous northward movement of the Indian plate [51]. As a result, the Hengduan Mountains gradually formed north–south mountain chains and large intervening fault zones (gorges), providing diverse habitats for species differentiation and becoming a diversity center [12, 17, 34]. The divergence rate of endemic genera in the Hengduan Mountains and East Himalaya regions increased during 15–10 Mya, with the rate being much higher in the East Himalaya region than in the Yunnan Plateau region, where it decreased. This suggests that the Hengduan Mountains and, in particular, the East Himalaya underwent a recent sharp uplift. Our findings indicate that the East Himalaya region was mainly characterized by temperate floristic elements, while the flora of the Yunnan Plateau region was predominantly tropical in nature, distinguishing it from the other two regions in the Sino-Himalaya [4, 55, 82].

## Conclusions

The assembly history of the angiosperm flora in the Sino-Himalaya can be categorized into four periods with different floristic elements. Our study indicated that prior to the Cenozoic, when the Sino-Himalaya was essentially a lowland bordered by the Tethys Sea, the flora was dominated by tropical elements. With the uplift of the Himalaya and adjacent areas, more temperate elements migrated in and diversified *in-situ*. We identified three major shifts in the divergence of genera and species during the four periods, which were likely influenced by geological events (such as the uplift of the Himalaya) and climate changes (such as the onset and intensification of the Asian monsoon system).

The temporal divergence patterns of tropical and temperate floristic elements varied across the three distinct floristic regions within the Sino-Himalaya. Historically, the Yunnan Plateau region predominantly exhibited tropical floristic elements, in contrast to the East Himalaya region, which was characterized mainly by temperate floristic elements. In the Hengduan Mountains region, however, temperate floristic elements surpassed tropical floristic elements from 30 Mya onwards. Moreover, the divergence rates of endemic genera and species in the Hengduan Mountains and East Himalaya regions considerably exceeded those in the Yunnan Plateau region over geological time. These findings suggest an asynchronous uplift of the Himalaya, the adjacent Yunnan Plateau, and the Hengduan Mountains, each occurring at distinct rates.

However, it should be noted that we might have overestimated the time of taxa emergence in the Sino-Himalayan flora because it is based on divergence time on the calibrated tree. Moreover, our study was constrained by the available data, as a dated phylogeny necessitates more precise fossil calibrations and a broader taxon sampling. To gain a more nuanced insight into the evolutionary history of the Sino-Himalayan flora, a comprehensive study integrating multiple disciplines at a granular level would be indispensable.

## Methods

### Species list and dated phylogeny

We obtained angiosperm species distribution data of the Sino-Himalayan flora from the dataset by Liu et al. [4] and compiled a list of angiosperm species in the Sino-Himalaya and its three floristic regions. Species names were standardized following the *Flora of China* [83]. The dated phylogeny of the Sino-Himalayan flora was obtained from the time-calibrated tree of angiosperms in the Sino-Himalaya and adjacent areas by Liu et al. [4]. The penalized likelihood method as implemented in treePL [84] (https://github.com/blackrim/treePL) was used to date the divergence times. A total of 123 fossil calibration points were used to estimate the divergence times of the taxa. Using the ‘ape’ package [85] in R 3.5.3 [86], we removed species from regions outside the Sino-Himalaya and obtained a time-calibrated tree composed of 8,260 (47%) species representing all families and 1,946 (90%) genera in the Sino-Himalayan angiosperm flora.

### Divergence time and statistical analyses of taxa

Using the above dated tree of the Sino-Himalayan angiosperm flora, we extracted the earliest possible times when families, genera, and species appeared in the flora. We followed the methodology of Lu et al. [45] for obtaining the divergence time of families, genera, and species in the Sino-Himalayan angiosperm flora. For monophyletic families and genera, stem ages were extracted directly by tracing their stem node. For families and genera that are polyphyletic or paraphyletic, the stem age of each monophyletic lineage was extracted, and the oldest one was selected as the age of the family and genus. Species divergence times were extracted from the ages of branch tips. We then calculated the number and percentage of families, genera, and species that diverged in each five-million-year period and different geological periods since the Cretaceous for the Sino-Himalaya and its three floristic regions.

### Classification and statistical analyses of floristic elements

The genera of the Sino-Himalayan flora were grouped into 15 distribution types based on Wu et al. [87] (Additional file 1: Table S5), which were later consolidated into three groups following Wu et al. [16]: distribution type 1 as widespread elements, distribution types 2–7 as tropical floristic elements, and distribution types 8–15 as temperate floristic elements. However, we found that some genera in distribution type 15 (Endemic to China) had tropical and subtropical characteristics. Therefore, we revised the classification of *Calcareoboea*, *Craspedolobium*, *Malania*, *Musella*, and *Whytockia* as temperate floristic elements. We determined the percentage of tropical floristic elements (*H*) and temperate floristic elements (*T*) that evolved in the Sino-Himalaya and its three floristic regions for each ten-million-year and geological period. The ratio of *H* to *T* (*HT*_ratio_) was calculated as follows:

$${HT}_{ratio}=\frac{(H-T)}{(H+T)}$$

*HT*_ratio_ < 0, temperate floristic elements are dominant in the studied period; *HT*_ratio_ > 0, tropical floristic elements are dominant in the studied period.

With the uplifts of the Himalaya and Hengduan Mountains, the temperate floristic elements in the Sino-Himalayan flora experienced significant development. We further classified the temperate floristic elements into three types: northern temperate (Wu’s distribution types 8–11), Tethys (12–13), and East Asian (14–15). We calculated the percentage of genera belonging to each of the four different floristic elements (e.g., tropical, northern temperate, Tethys, and East Asian) that evolved in the Sino-Himalayan flora during each geological period.

### Statistical analyses of endemic genera and endemic species

We obtained the list of endemic genera of the Sino-Himalayan angiosperm flora (Additional file 2) by extracting the genera with distribution types 14 and 15 that were confined to the Sino-Himalaya and adjacent areas, and the distribution information of the genera was obtained from the *Flora of China* [83]. Endemic species were defined as those with 90% of their distribution range being restricted to the Sino-Himalaya. The list of endemic species was based on the inventory of Chinese endemic seed plant species from Huang et al. [88] and county-level species distribution information from the dataset of Lu et al. [45] (Additional file 2). We then extracted the records of endemic genera and species separately for the three floristic regions within the Sino-Himalaya.

Statistical analyses were conducted on the number and percentage of endemic genera and species that diverged in the Sino-Himalaya and its three floristic regions during each five-million-year period and geological period.

### Supplementary Information

**Additional file 1: Fig. S1.** Statistics of angiosperm families, genera, and species diverged over geological time in the three floristic regions (i.e., the Yunnan Plateau region, Hengduan Mountains region, and East Himalaya region) within the Sino-Himalaya. (a), (b) families; (c), (d) genera; (e), (f) species. The lines with different colors represent the number of taxa that diverged during each five-million-year period in different floristic regions. The bar charts with different colors represent the percentages of taxa that diverged during each geological period in different floristic regions. EC, Early Cretaceous; LC, Late Cretaceous; PA, Paleocene; EO, Eocene; OL, Oligocene; MI, Miocene; PL, Pliocene; Qu, Quaternary. **Fig. S2.** Statistics of each distribution type (1–15) of genera in the Sino-Himalayan flora that diverged during each geological period. (a) Percentage of genera with each distribution type; (b) percentage of genera with each distribution type that diverged during each geological period; (c) changes in the percentage of genera with each distribution type over geological time. The 15 distribution types of genera were documented according to Wu et al. [87] (Additional file 1: Table S5). PA, Paleocene; EO, Eocene; OL, Oligocene; MI, Miocene; PL, Pliocene; Qu, Quaternary. **Fig. S3.** Statistics of tropical and temperate floristic elements that evolved during each geological period in the Sino-Himalaya and its three floristic regions. (a) Sino-Himalaya; (b) Yunnan Plateau region; (c) Hengduan Mountains region, and (d) East Himalaya region. The red bar charts represent the number of diverged genera with tropical floristic elements, i.e., distribution types 2–7 in Wu et al. [87]; the blue bar charts represent the number of diverged genera with temperate floristic elements, i.e., distribution types 8–14 in Wu et al. [87]. The red and blue lines represent the percentages of diverged genera with tropical and temperate floristic elements during each geological period, respectively. PA, Paleocene; EO, Eocene; OL, Oligocene; MI, Miocene; PL, Pliocene; Qu, Quaternary. **Fig. S4.** Divergence patterns of four floristic elements (i.e., the tropical, Tethys, northern temperate, and East Asia floristic elements) of angiosperm genera in the Sino-Himalayan flora over geological time. (a) Percentages of diverged genera with four floristic elements; (b) percentage of diverged genera with each distribution type of the tropical floristic elements; (c) percentage of diverged genera with each distribution type of the Tethys floristic elements; (d) percentage of diverged genera with each distribution type of the northern temperate floristic elements; (e) percentage of genera with each distribution type of the East Asia floristic elements. Distribution types of genera were documented according to Wu et al. [87] (Additional file 1: Table S5). EC, Early Cretaceous; LC, Late Cretaceous; PA, Paleocene; EO, Eocene; OL, Oligocene; MI, Miocene; PL, Pliocene; Qu, Quaternary. **Table S1.** Numbers and percentages of angiosperm families that may have diverged during each geological period in the Sino-Himalaya and its three floristic regions. **Table S2.** Number and percentages of angiosperm genera that may have diverged during each geological period in the Sino-Himalaya and its three floristic regions. **Table S3.** Number and percentages of angiosperm species that may have diverged during each geological period in the Sino-Himalaya and its three floristic regions. **Table S4.** Number and percentages of angiosperm genera of four floristic elements that may have diverged during each formation period in the Sino-Himalayan flora.** Table S5.** Distribution types of genera of the Chinese flora according to Wu et al. [87].

**Additional file 2. **List of endemic genera and species of the Sino-Himalayan flora.

## Data Availability

All data generated or analysed during this study are included in this published article and its supplementary information files.

## Abbreviation

Mya: Million years ago
