# Supplementary material for: The Sino-Himalayan flora evolved from lowland biomes dominated by tropical floristic elements
Source: BMC Biol. 2023 Oct 31;21:239. doi: 10.1186/s12915-023-01746-4 (PMC10617089; doi:10.1186/s12915-023-01746-4)
Supplement: Supplementary file 1 — Additional file 1: Fig. S1. Statistics of angiosperm families, genera, and species diverged over geological time in the three floristic regions (i.e., the Yunnan Plateau region, Hengduan Mountains region, and East Himalaya region) within the Sino-Himalaya. (a), (b) families; (c), (d) genera; (e), (f) species. The lines with different colors represent the number of taxa that diverged during each five-million-year period in different floristic regions. The bar charts with different colors represent the percentages of taxa that diverged during each geological period in different floristic regions. EC, Early Cretaceous; LC, Late Cretaceous; PA, Paleocene; EO, Eocene; OL, Oligocene; MI, Miocene; PL, Pliocene; Qu, Quaternary. Fig. S2. Statistics of each distribution type (1–15) of genera in the Sino-Himalayan flora that diverged during each geological period. (a) Percentage of genera with each distribution type; (b) percentage of genera with each distribution type that diverged during each geological period; (c) changes in the percentage of genera with each distribution type over geological time. The 15 distribution types of genera were documented according to Wu et al. [87] (Additional file 1: Table S5). PA, Paleocene; EO, Eocene; OL, Oligocene; MI, Miocene; PL, Pliocene; Qu, Quaternary. Fig. S3. Statistics of tropical and temperate floristic elements that evolved during each geological period in the Sino-Himalaya and its three floristic regions. (a) Sino-Himalaya; (b) Yunnan Plateau region; (c) Hengduan Mountains region, and (d) East Himalaya region. The red bar charts represent the number of diverged genera with tropical floristic elements, i.e., distribution types 2–7 in Wu et al. [87]; the blue bar charts represent the number of diverged genera with temperate floristic elements, i.e., distribution types 8–14 in Wu et al. [87]. The red and blue lines represent the percentages of diverged genera with tropical and temperate floristic elements during each geological peri [file 12915_2023_1746_MOESM1_ESM.docx]

**Fig. S1**


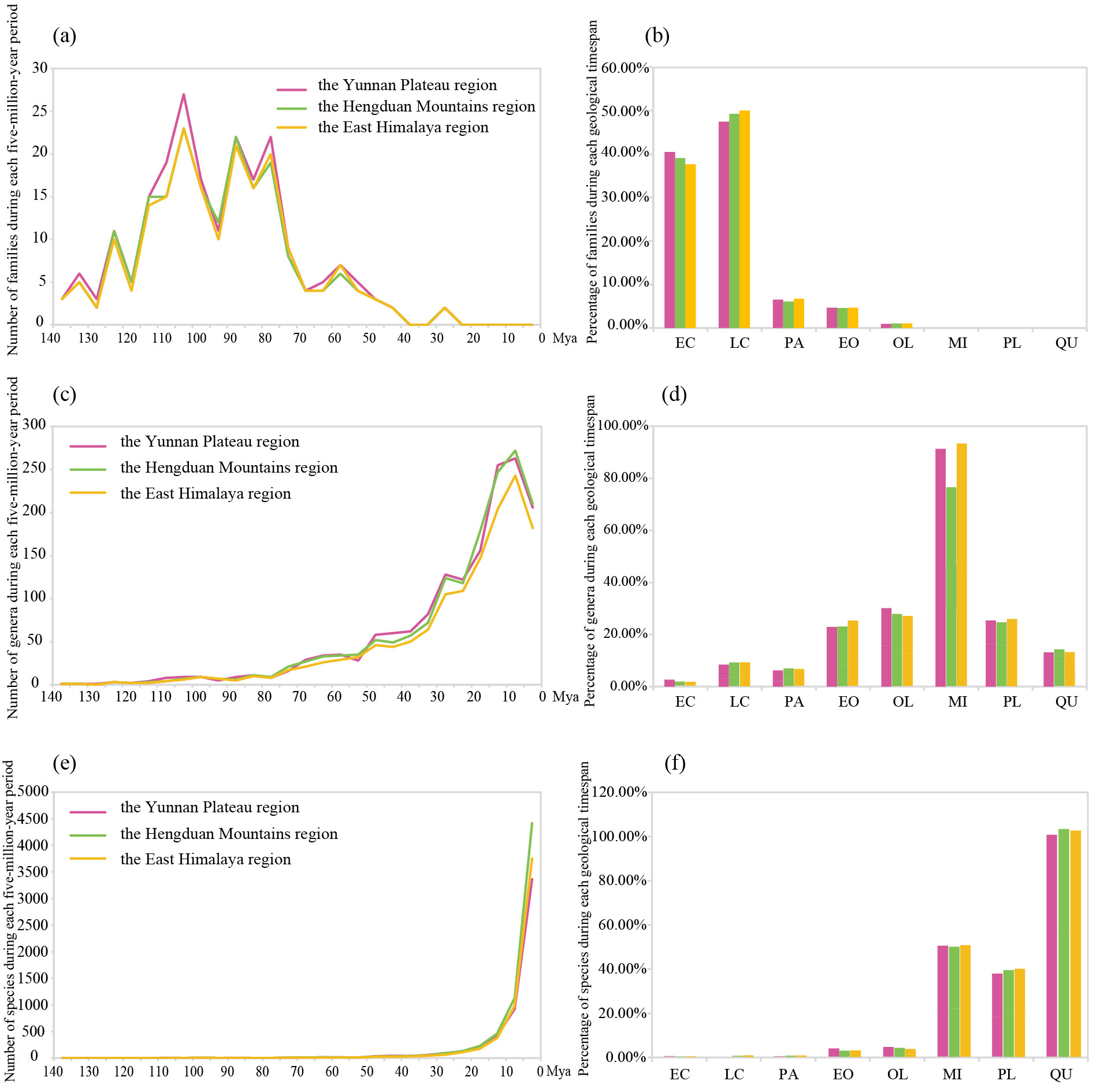


**Fig. S2**

**
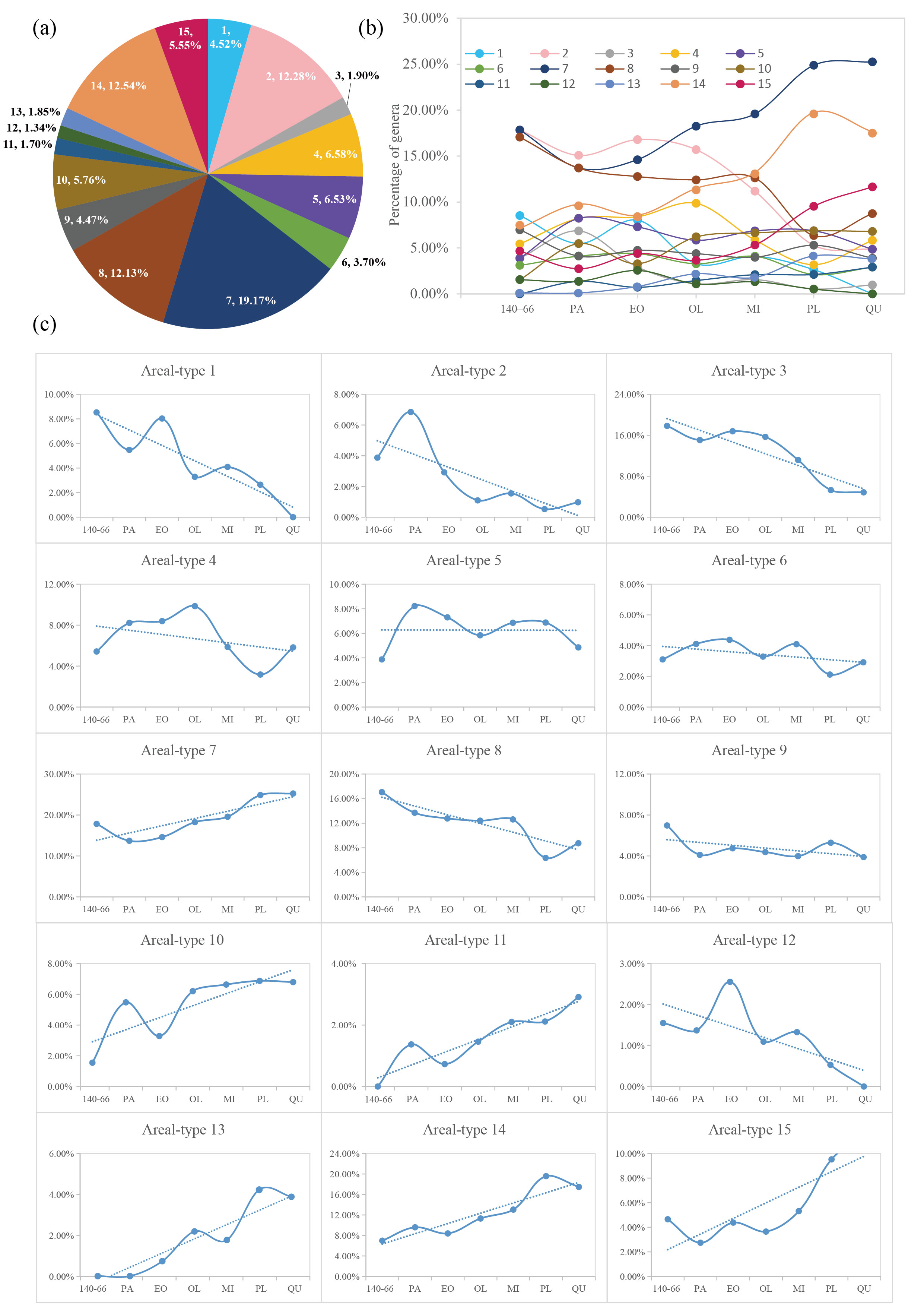
**

**Fig. S3**

**
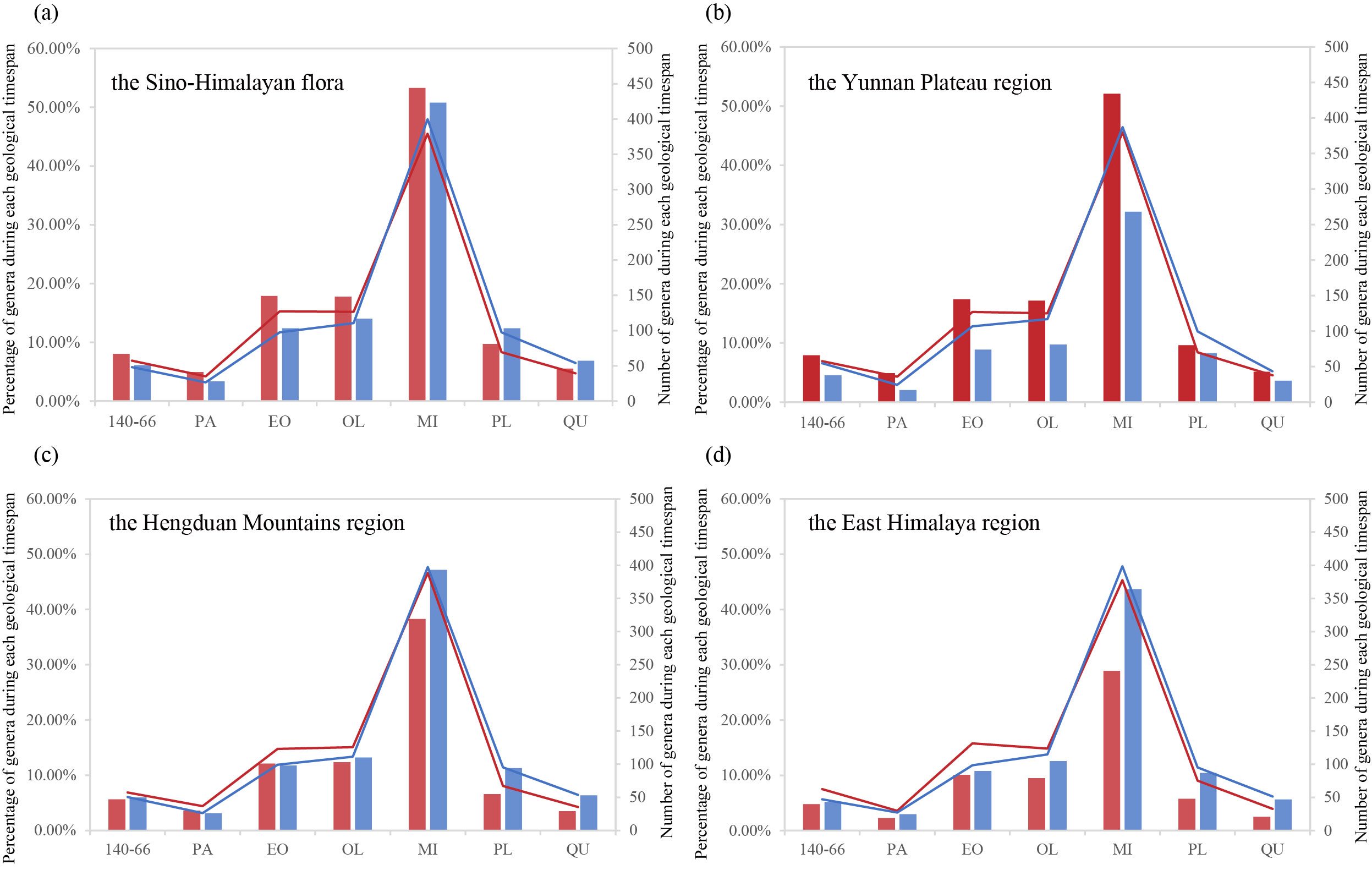
**

**Fig. S4**


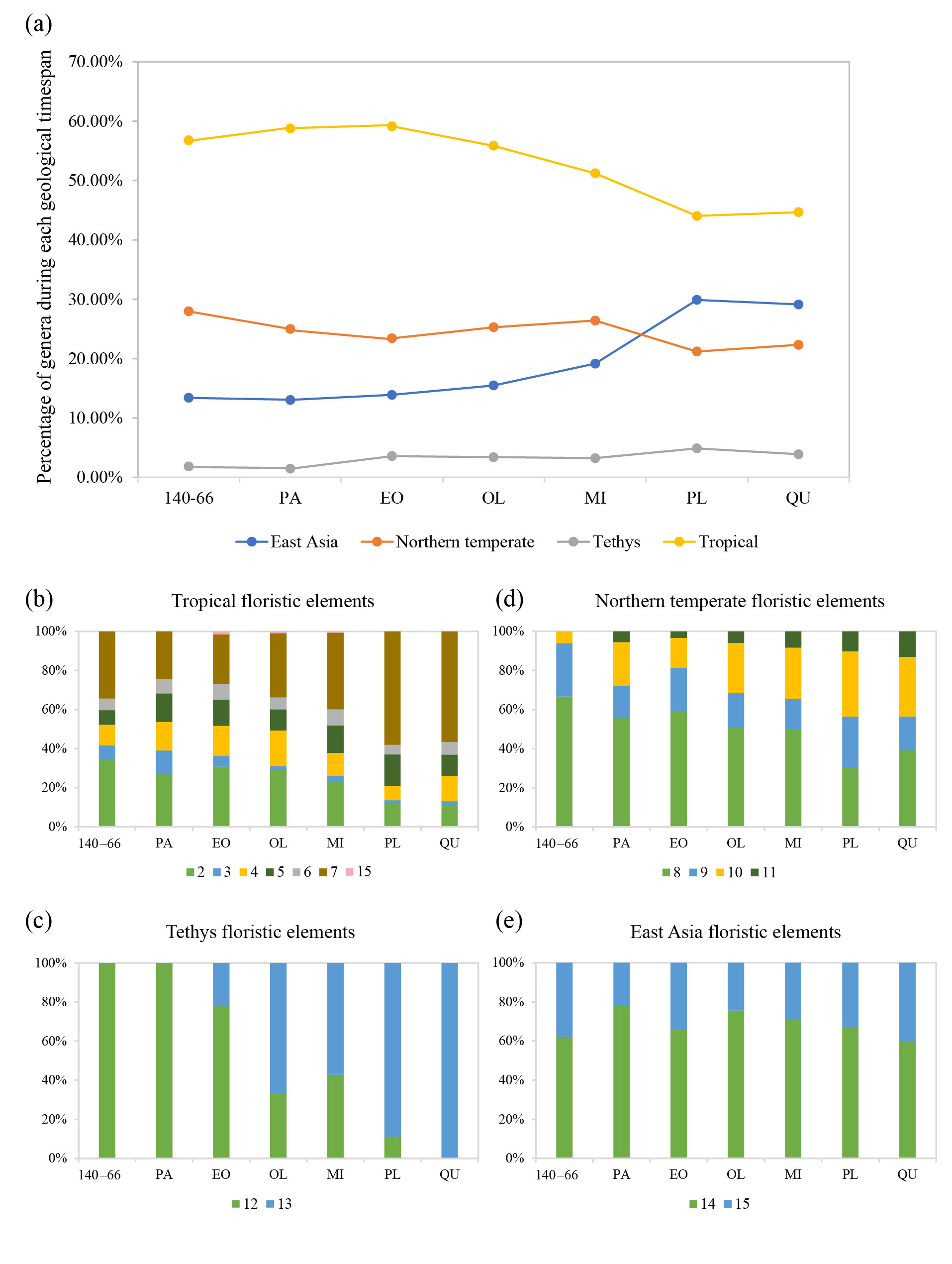


**Table S1**

| Geological period | the Sino-Himalayan flora | the Yunnan Plateau region | the Hengduan Mountains region | the East Himalaya region |
| --- | --- | --- | --- | --- |
| Early Cretaceous | 89  (40.09%) | 87  (40.47%) | 77  (39.09%) | 73  (37.63%) |
| Late Cretaceous | 107  (48.20%) | 102  (47.44%) | 97  (49.24%) | 97  (50.00%) |
| Paleocene | 14  (6.31%) | 14  (6.51%) | 12  (6.09%) | 13  (6.70%) |
| Eocene | 10  (4.50%) | 10  (4.65%) | 9  (4.57%) | 9  (4.64%) |
| Oligocene | 2  (0.90%) | 2  (0.93%) | 2  (1.02%) | 2  (1.03%) |
| Miocene | 0 | 0 | 0 | 0 |
| Pliocene | 0 | 0 | 0 | 0 |
| Pleistocene | 0 | 0 | 0 | 0 |
| All | 222 | 215 | 197 | 194 |

**Table S2**

| Geological period | the Sino-Himalayan flora | | the Yunnan Plateau region | | | the Hengduan Mountains region | | | the East Himalaya region | |  |
| --- | --- | --- | --- | --- | --- | --- | --- | --- | --- | --- | --- |
|  | all | endemic | | all | endemic | all | endemic | all | | endemic | |
| Early Cretaceous | 30  (1.54%) | 1  (0.69%) | | 29  (1.81%) | 1  (1.19%) | 21  (1.32%) | 1  （0.76%） | 18  (1.31%) | | 1  (0.74%) | |
| Late Cretaceous | 99  (5.09%) | 4  (2.76%) | | 84  (5.23%) | 3  (3.57%) | 86  (5.40%) | 4  (3.03%) | 75  (5.45%) | | 4  (2.96%) | |
| Paleocene | 73  (3.75%) | 4  (2.76%) | | 61  (3.80%) | 2  (2.38%) | 61  (3.83%) | 4  (3.03%) | 48  (3.49%) | | 4  (2.96%) | |
| Eocene | 274  (14.08%) | 15  (10.34%) | | 237  (14.77%) | 9  (10.71%) | 220  (13.81%) | 13  (9.85%) | 194  (14.09%) | | 15  (11.11%) | |
| Oligocene | 274  (14.08%) | 18  (12.41%) | | 233  (14.52%) | 13  (15.48%) | 220  (13.81%) | 17  (12.88%) | 193  (14.02%) | | 17  (12.59%) | |
| Miocene | 904  (46.45%) | 67  (46.21%) | | 734  (45.73%) | 34  (40.48%) | 749  (47.02%) | 60  (45.45%) | 641  (46.55%) | | 61  (45.19%) | |
| Pliocene | 189  (9.71%) | 23  (15.86%) | | 154  (9.60%) | 13  (15.48%) | 154  (9.67%) | 20  (15.15%) | 140  (10.17%) | | 21  (15.56%) | |
| Pleistocene | 103  (5.29%) | 13  (8.97%) | | 73  (4.55%) | 9  (10.71%) | 82  (5.15%) | 13  (9.85%) | 68  (4.94%) | | 12  (8.89%) | |
| All | 1946 | 145 | | 1605 | 84 | 1593 | 132 | 1377 | | 135 | |

**Table S3**

| Geological period | the Sino-Himalayan flora | | the Yunnan Plateau region | | the Hengduan Mountains region | | | the East Himalaya region | | |
| --- | --- | --- | --- | --- | --- | --- | --- | --- | --- | --- |
|  | all | endemic | all | endemic | all | endemic | all | | endemic |  |
| Early Cretaceous | 11  (0.13%) | 0 | 10  (0.19%) | 0 | 7  (0.10%) | 0 | 6  (0.11%) | | 0 |  |
| Late Cretaceous | 45  (0.54%) | 0 | 32  (0.60%) | 0 | 34  (0.51%) | 0 | 25  (0.44%) | | 0 |  |
| Paleocene | 38  (0.46%) | 2  (0.16%) | 28  (0.52%) | 0 | 25  (0.37%) | 1  (0.09%) | 19  (0.34%) | | 2  (0.20%) |  |
| Eocene | 187  (2.26%) | 9  (0.74%) | 147  (2.74%) | 6  (1.31%) | 134  (2.00%) | 5  (0.47%) | 109  (1.93%) | | 6  (0.61%) |  |
| Oligocene | 222  (2.69%) | 11  (0.91%) | 167  (3.11%) | 5  (1.09%) | 169  (2.52%) | 11  (1.03%) | 123  (2.18%) | | 9  (0.92%) |  |
| Miocene | 2281  (27.62%) | 268  (22.09%) | 1524  (28.39%) | 102  (22.27%) | 1779  (26.58%) | 235  (22.11%) | 1502  (26.57%) | | 222  (22.68%) |  |
| Pliocene | 1599  (19.36%) | 239  (19.70%) | 1047  (19.50%) | 92  (20.09%) | 1326  (19.81%) | 210  (19.76%) | 1128  (19.96%) | | 193  (19.71%) |  |
| Pleistocene | 3877  (46.94%) | 684  (56.39%) | 2413  (44.95%) | 253  (55.24%) | 3220  (48.10%) | 601  (56.54%) | 2740  (48.48%) | | 547  (55.87%) |  |
| All | 8260 | 1213 | 5368 | 458 | 6694 | 1063 | 5652 | | 979 |  |

**Table S4**

| Period | the tropical floristic elements | the temperate floristic elements | | |
| --- | --- | --- | --- | --- |
|  | the tropical floristic elements | the Tethys floristic elements | the northern temperate floristic elements | the East Asia floristic elements |
| I | 108  (57.75%) | 3  (1.60%) | 51  (27.27%) | 25  (13.37%) |
| II | 300  (58.03%) | 18  (3.48%) | 126  (24.37%) | 73  (14.12%) |
| III | 446  (51.44%) | 28  (3.23%) | 229  (26.41%) | 164  (18.92%) |
| IV | 127  (44.25%) | 13  (4.53%) | 62  (21.60%) | 85  (29.62%) |
| All | 981  (52.80%) | 62  (3.34%) | 468  (25.19%) | 347  (18.68%) |

**Table S5**

| Distribution types | Distribution types |
| --- | --- |
| 1 | Cosmopolitan |
| 2 | Pantropic |
| 3 | Tropical Asia and Tropical America disjuncted |
| 4 | Old World Tropics |
| 5 | Tropical Asia and Tropical Australia |
| 6 | Tropical Asia and Tropical Africa |
| 7 | Tropical Asia (Indo-Malaysia) |
| 8 | North Temperate |
| 9 | East Asia and North America disjuncted |
| 10 | Old World Temperate |
| 11 | Temperate Asia |
| 12 | Mediterranean, West Asia to Central Asia |
| 13 | Central Asia |
| 14 | East Asia |
| 15 | Endemic to China |
